# Supplementary material for: Improving diagnosis and prognosis of lung cancer using vision transformers: a scoping review
Source: BMC Med Imaging. 2023 Sep 15;23:129. doi: 10.1186/s12880-023-01098-z (PMC10503208; doi:10.1186/s12880-023-01098-z)
Supplement: Supplementary file 2 — Appendix 2: Search strings [file 12880_2023_1098_MOESM2_ESM.docx]

**Appendix 2: Search strings**

Database(s): IEEE Xplore, Pubmed, Scopus and Google Scholar.

Years: 2017-2022

Search dates: 21 December 2022

Initial raw search results: 314

| **Database** | **Search strategy** | **Hits** |
| --- | --- | --- |
| IEEExplore | ((Transformer) OR (Vision Transformer)) AND ((Lung Cancer) OR (lung nodule) OR (carcinoma) OR (Adenocarcinoma) OR (Pancoast) OR (Carcinoid)) | 19 |
| Pubmed | ((Transformer) OR (Vision Transformer)) AND ((Lung Cancer) OR (lung nodule) OR (carcinoma) OR (Adenocarcinoma) OR (Pancoast) OR (Carcinoid)) | 49 |
| Scopus | TITLE-ABS-KEY ( ( ( transformer )  OR  ( vision  AND  transformer ) )  AND  ( ( lung  AND  cancer )  OR  ( lung  AND  nodule )  OR  ( carcinoma )  OR  ( adenocarcinoma )  OR  ( pancoast )  OR  ( carcinoid ) ) )  AND  ( LIMIT-TO ( PUBYEAR ,  2023 )  OR  LIMIT-TO ( PUBYEAR ,  2022 )  OR  LIMIT-TO ( PUBYEAR ,  2021 )  OR  LIMIT-TO ( PUBYEAR ,  2020 )  OR  LIMIT-TO ( PUBYEAR ,  2019 )  OR  LIMIT-TO ( PUBYEAR ,  2018 ) ) | 96 |
| Google Scholar | ((Transformer) OR (Vision Transformer)) AND ((Lung Cancer) OR (lung nodule) OR (carcinoma) OR (Adenocarcinoma) OR (Pancoast) OR (Carcinoid)) | 150 |
