# Supplementary material for: Improving diagnosis and prognosis of lung cancer using vision transformers: a scoping review
Source: BMC Med Imaging. 2023 Sep 15;23:129. doi: 10.1186/s12880-023-01098-z (PMC10503208; doi:10.1186/s12880-023-01098-z)
Supplement: Supplementary file 3 — Appendix 3: Data extraction form [file 12880_2023_1098_MOESM3_ESM.docx]

**Appendix 3: Data extraction form**

| **Concept** | **Definition** |
| --- | --- |
| **Study Characteristics** |  |
| ID | Unique ID assigned to each study |
| Author | The first author of the study. |
| Year | The year in which the study was published. |
| Month | Month of publication. |
| Country of publication | Affiliation of the first author of the study. |
| Venue name | Name of the conference or journal where the study was published. |
| Publication type | Journal or conference or book chapter. |
| **Model** |  |
| 3D Model | Model for volumetric data. |
| 2D Model | Model for two-dimensional data. |
| Modality of data | Image modality such as CT, X Ray, Histopathology, MRI. |
| Purpose or aim | Main task addressed in the study, such as classification, survival prediction, prognosis or segmentation. |
| Comments on architecture | Brief comments on the artchitecture of the model. For example, vision transformers combined with CNN or UNet or GAN, etc. |
| Transformer name | Specific variant of the transformer (if available), such as SWIN transformer. |
| **Implementation** |  |
| Implementation framework | Implementation framework such as Pytorch, Tensorflow, or JAX |
| Hardware resources | Information related to the use of computational resources such as GPUs. |
| **Dataset** **Characteristics** |  |
| Image modality | Type of imaging modality used in the study, such as MRI, Pathology, etc. |
| Data availabilitiy | Availability of data as public or private dataset. |
| Name of the data | The specific name of the dataset, if available. |
| Data source (full URL) | Mention full URL of the dataset |
| Dataset size (Number of subjects or individuals) | For how many individuals the data is recorded? |
| Dataset size (number of images) | What is the total number of images in the dataset? |
| Training set | What is the number of images or subjects in the training set? |
| Validation set size | What is the number of images or subjects in the validation set? |
| Testing set size | What is the number of images or subjects in the test set? |
| **Evaluation** |  |
| Type of validation | What type of validation the authors used? (e.g., train test split, k-fold cross validation, external validation) |
| Evaluation metrics | Metrics of evaluation (for example, Accuracy, Dice similarity coefficient, AUCROC, F1 score, Concordance index) |
| Study aim | One statement summary of the work. |
| Code URL | Full URL of the software code, if available. |
